# Patient experiences of primary care quality amongst different types of health care facilities in central Vietnam

**DOI:** 10.1186/s12913-019-4089-y

**Published:** 2019-05-02

**Authors:** Nguyen Thi Hoa, Nguyen Minh Tam, Anselme Derese, Jeffrey F. Markuns, Wim Peersman

**Affiliations:** 1 Department of Family Medicine, Hue University of Medicine and Pharmacy, Hue University, 06 Ngo Quyen street, Hue, Vietnam; 2 Department of Public Health and Primary Care, Campus UZ 6K3, C.Heymanslaan 10, B-9000 Ghent, Belgium; 3 Global Health Collaborative, Department of Family Medicine, Boston University, Boston, MA 02118 USA; 4 Research Group Social Work, Odisee University College, Warmoesberg 26, B-1000 Brussel, Belgium; 5 Department of Rehabilitation Sciences, Ghent University, Campus UZ, C. Heymanslaan 10, B-9000 Ghent, Belgium

**Keywords:** Primary care, Patient experiences, Health care settings, PCAT, Vietnam

## Abstract

**Background:**

Patient experience with primary health care services can vary markedly between different types of health care facilities, even within the same country setting. Given known benefits of high quality primary health care, the performance of these facilities may significantly impact population health. The aim of this study was to compare the quality of primary care in different types of health facilities as experienced by Vietnamese consumers.

**Methods:**

1662 people who utilized primary health care services at least once over the past two years in various types of facilities in central Vietnam were surveyed in a cross-sectional study using the Vietnamese version of the Primary Care Assessment Tool (VN PCAT-AE) to assess overall primary care quality as well as several different domains of high quality primary care services.

**Results:**

Commune health centers were associated with the highest overall primary care quality (PCAT expanded score 21.07, *p* < 0.001) as well as high scores in nearly all individual domains of primary care quality experienced by consumers compared with other types of facilities. Conversely, private facilities such as private clinics and pharmacies were rated lowest overall (PCAT expanded score 18.45, *p* < 0.05 and 16.90, p < 0.001 respectively). District hospitals and other government hospitals (PCAT expanded score 20.10 and 19.72 respectively) were reported as the best quality in comprehensiveness of available services (p < 0.001). Polyclinics performed quite well in comprehensiveness of services available (3.11) and first contact-access (2.79) but less so in other domains, especially in cultural competency (1.87).

**Conclusions:**

The high quality of primary care services experienced by consumers in commune health centers compared with other facilities gives Vietnam ample reason to promote greater use of these community-based primary care facilities. Populations may benefit most from building and strengthening grassroots networks of such community-based health centers as an effective solution for overcrowding at hospitals while simultaneously providing better overall health outcomes.

**Electronic supplementary material:**

The online version of this article (10.1186/s12913-019-4089-y) contains supplementary material, which is available to authorized users.

## Background

Primary care has been shown to result in better health outcomes for populations, and is a cornerstone for the types of improvements sought in the new Sustainable Development Goal for Health [1]. A strong primary care system is essential to providing effective and efficient health care in all countries and has been correlated to lower aggregate and gender-specific mortality rates, overall levels of premature death, and premature deaths from a variety of important preventable or treatable conditions including asthma, heart and cerebrovascular diseases, and pneumonia [2, 3]. The structure of local health care systems and their associated facilities, however, can have a substantial impact on the accessibility, acceptability, effectiveness and quality of primary care. In the early work by quality advocates, Donabedian also laid out that given the proper settings and instrumentalities, good medical care will follow but the relationship between structure and outcome, is often not well established [4].

Consensus is now building around a comprehensive framework that highlights the important role of facilities in primary health care systems as well as the need to assess these based on specific elements associated with high quality primary care service delivery [5]. The quality of patient experiences with primary care can vary markedly between different health care facilities, even within the same country setting. Studies in the U.S., Hong Kong and China have all shown some impact of different types of facilities on the quality of primary care provided [6–13]. Although evidence from low and middle income countries shows that an integrated approach to primary care can improve health outcomes, less is known about the quality of primary care provided by different types of facilities in these lower income countries [14].

Vietnam has a tiered health system, with a variety of different health facilities where patients can directly seek primary care services. At the grassroots level, there is a widespread public system of more than 11,000 commune health centers (CHCs), one in every commune. The commune health center (CHC) has the capacity to deliver preventive, acute and chronic care, and treatment services for individuals as well as for families in each commune [15]. Most CHCs are staffed with a general doctor and ancillary staff, and typical services include immunization, epidemic prevention, first aid, maternal and child health care, and treatment of common health problems such as chronic and infectious diseases. In addition to CHCs, there are district health center-operated outpatient polyclinics, staffed by physicians from a variety of specialties which offer diagnostic and treatment services for a range of health problems. A polyclinic provides health care for a number of nearby communes in a region, supplementing local CHC activities. As a next step up in the tiered public health care system, district health centers (DHC) in every district provide more complex curative services and typically include an outpatient department for diagnostic and therapeutic services. They also receive patients who are referred by CHCs in the local region, as district health centers offer more diagnostic services as well as an inpatient departments, with disciplines such as internal medicine, paediatrics, surgery, obstetrics and gynaecology.

Additional hospital levels beyond the districts include a provincial hospital in each province and central hospitals in each region, again typically offering a variety of more complex inpatient and outpatient services. However, in the joint annual health review JAHR 2015 of the Ministry of Health [16], data showed that 54–65% of patients coming to central hospitals have diseases and health conditions that are diagnosable and treatable at the lower levels. This is one challenge faced by policy-makers in Vietnam, leading to the overcrowding of upper level public hospitals because many patients bypass the grassroots outpatient facilities, and even the district health centers, as patients expect a better quality of care in these more advanced hospitals [17]. Similar to hospitals, patients may pursue private sector services seeking what they perceive as higher quality care, such as in the private clinics of prominent clinicians. Patients also frequent a variety of other health facilities when seeking primary care services, such as basic advice and accompanying therapeutics from the local pharmacy.

While Vietnam is working towards universal health coverage, it has yet to be achieved. Coverage rates for health insurance in Vietnam have increased since its introduction in 1992 to 71% in 2014 and nearly 88% by 2018 [18]. There are two types of government-run health insurance schemes with all individuals categorized into either compulsory or voluntary health insurance. Compulsory insurance is offered to contracted employed workers, elderly, children under age six, students and the poor with the remainder eligible for voluntary insurance. Because of the limited coverage amounts subsidized by the government as well as the voluntary health insurance scheme, however, some patients do still experience financial barriers to access. Full coverage for services of insured patients is available if patients initially seek health care at a grassroots facility (i.e. CHC, polyclinic), however a payment is required if they present to a district or provincial hospital without a referral letter from the previous level health facility.

Like many other countries in the world searching for an ideal model of primary care delivery, Vietnam has been conducting a national program for reinforcement and quality improvement of primary care focusing on the grassroots level using a variety of public and private services [19–21]. Little is known, however, about the difference in primary care quality in different health care settings in Vietnam. We conducted this study with the hypothesis that the quality of primary health care as experienced by consumers would differ between the various types of facilities. Our hypothesis was that those facilities whose primary function was to deliver grassroots health care and act as a first point of entry to the health system, such as community-based government run health centers, would be rated more highly by consumers on primary care quality compared with those facilities with other primary functions such as hospitals focused on secondary and tertiary care, private sector pharmacies focused on market-based provision of medications, or private clinics may have a focus on the specific medical area of a particular specialist.

## Methods

### Aim

The aim of this study was to compare the quality of primary care in different types of health facilities as experienced by Vietnamese consumers.

### Study population and design

**Fig. 1:**
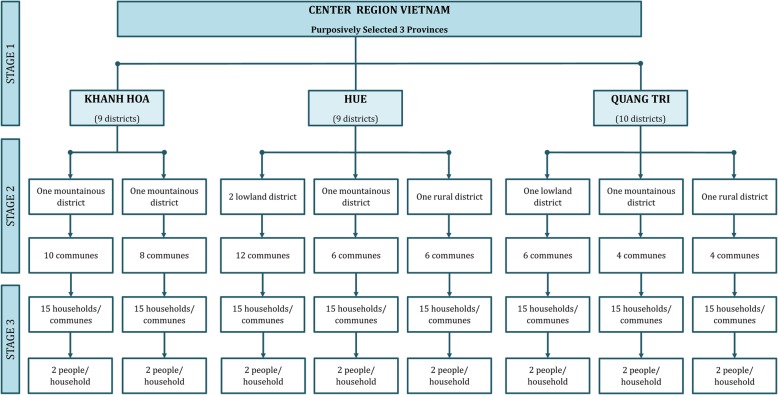
Multistage sampling method

This cross-sectional study was conducted in the central region of Vietnam using a multistage and purposive sampling approach (illustrated in Fig. 1). Three provinces were chosen purposively to capture the diverse characteristics of central Vietnam: Khanh Hoa, Thua Thien Hue and Quang Tri. To obtain a sample representing the country’s diversity, we purposively selected two to four districts from each province. Within these constraints, we chose at least one lowland district, one mountainous district and one urban district when possible. In Thua Thien Hue, the study was carried out in 24 communes of four districts (six communes per district); in Quang Tri, 14 communes in three districts (one district with six communes and two other districts with four communes); and in Khanh Hoa, 18 communes in two districts. In total, 56 communes in 9 districts were selected. Additional file 1 shows the locations map of these districts and communes.

From each commune, 15 households were selected from the commune household list. On the list, we started with the first household of the commune and then selected every 10th household (household number 11, 21, 31…) until the intended sample size was reached. Each selected household was visited, and the head of household surveyed, as well as one other willing adult (≥18 years old) if available during this home visit. Data was collected from January through August of 2014 and questionnaires were administered through in-person interviews. Only participants who had utilized health care services at a health facility at least once within the two years prior to recruitment were surveyed.

Before the interview, participants received a full explanation of the study’s content and purpose and signed a consent form if they agreed to participate. Refusals were rare and so a response rate was not specifically tracked, but surveyors estimated the combined refusal and non-response rates at less than 5%. If a household refused or could not be reached after three attempts, then another household was chosen at random from the reserve list. Participants were compensated for their time with small gifts of appreciation (worth $2.50 USD) upon completion of the interview.

Surveyors are volunteer medical students of local medical universities and colleges who are living in the study area. Surveyors received training courses on the purpose and content of the research project as well as interviewing skills in the month before data collection was conducted. There were always two research team members supervising the data collection per location. Supervisors and interviewers met together every evening when interviewers finished their field work to review the process of that day.

### Study materials

This study used an adaptation of the adult consumer expanded version of the Primary Care Assessment Tool (PCAT-AE) originally developed at Johns Hopkins University [22]. This tool was designed to assess primary care quality by measuring key primary care domains based on the experience of consumers. Versions of the original tool have been validated and commonly applied around the world successfully [23–26]. The Vietnamese version of this tool (VN PCAT-AE) was validated and demonstrated adequate internal consistency and validity with this study population as a tool for measuring the quality of primary care in Vietnam [27].

**Fig. 2:**
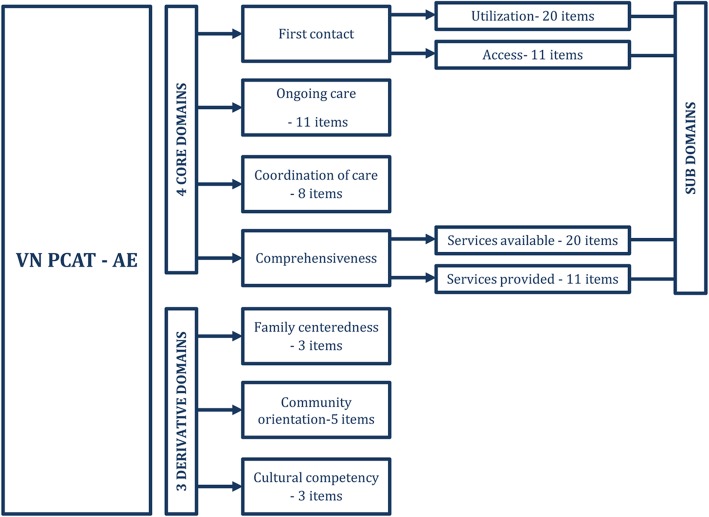
VN PCAT-AE and its domains

Retaining most of the major characteristics of the original version with 70 items (see Fig. 2), the VN PCAT-AE has six scales representing four core primary care domains: 1) first contact with two subdomains: accessibility (three items) and utilization (six items), 2) ongoing care (11 items), 3) coordination (eight items), and 4) comprehensiveness of services with two subdomains: available services (20 items) and services provided (11 items). It also includes three other scales representing three derivative domains: 1) family centeredness (three items), 2) community orientation (five items) and 3) cultural competence (three items). Except for the scale of Family Centeredness (0.68), all of the scales have a Cronbach’s alpha above 0.70 [27].

The VN PCAT-AE uses a 4-point Likert scale response (1 = definitely not; 2 = probably not; 3 = probably; and 4 = definitely) and an additional “don’t know/don’t remember” option for each item. The recoding process and calculation for the sum mean score of domains and subdomains as well as the total primary care score (PCAT score) and the total primary care expanded score (PCAT expanded score) strictly complied with the guideline PCAT manual issued by John Hopkins University [28]. The score of each domain and subdomain is the mean of sum scores of all items within each. The total primary care score (PCAT score) quantifies primary care quality using the sum mean scores of the six subdomains in the four core domains. The total primary care expanded score (PCAT expanded score) is the sum mean scores of the nine core and derivative subdomains. For calculating the sum mean scores, a mean value was assigned to “don’t know/don’t remember” answers as well as to missing values.

Three questions were used to inquire about an individual’s usual source of care as a particular person or place [22]. For those with no identifiable source of primary care, subsequent questions were asked about the last place that was visited.

The questionnaire also included questions about demographic characteristics such as age, gender, occupation, living area, as well as health condition of participants.

### Data analysis

All collected questionnaires were cleaned and scanned into a computer for storage and convenient review in the future, followed by entry into EpiData by a group of six students working in pairs. Double data entry was used to check for errors in data entry. The SPSS convert file was used to check for errors due to incorrect data entry. The Chi-square test was used to test for differences in the demographic characteristics of consumers from different types of health care facilities. ANOVA was conducted for comparison on scores of each primary care attribute, the PCAT score and the PCAT expanded score between health care settings after adjusting for gender, age, education level, job status, living area, chronic health problems, health insurance coverage and time affiliation with health facilities. Differences in the means of adjusted scores between health care settings were also tested using pair-wise comparison with the Bonferroni post hoc test for multiple-testing [29]

## Results

### Characteristic of study participants

Our study population consisted of 1662 adults, living in the central region of Vietnam, who visited a health facility at least once within the two years prior to recruitment. Table 1 shows the health facility choices of survey respondents. In general, commune health centers (CHCs) were the most common choice of respondents (39.6%, *n* = 658). In contrast, private clinics (PVC) were only rarely used (7.8%, *n* = 129). Utilization of polyclinics (PLC), district health centers (DHC), and pharmacies (PHM) as a usual source of primary care was about 12–14% for each of these categories. All higher-level government hospitals combined (GVH) such as provincial hospitals, central hospitals or other hospitals including university, military or traditional medicine hospitals were utilized as a source of primary care at a similar rate to the other alternatives to CHCs (15.3%, *n* = 255).

**Table 1:** Socio-demographic and health related characteristics of participants by type of health facility

Variable		CHC	PLC	DHC	PVC	PHM	GVH	p
		n (%)	n (%)	n (%)	n (%)	n (%)	n (%)	
N (%)		658 (39.6)	196	228(13.7)	129(7.8)	196(11.8)	255(15.3)	
Gender N = 1662	Male	298(40.2)	80(10.8)	111(15.0)	46(6.2)	78(10.5)	128(17.3)	0.034
	Female	360(39.1)	116(12.6)	117(12.7)	83(9.0)	118(12.8)	127(13.8)	
Age N = 1660	18 to 39-year-old	218(49.5)	38(8.6)	42(9.5)	38(8.6)	54(12.3)	50(11.4)	< 0.001
	40 to 59-year-old	275(35.4)	100(12.9)	124(16.0)	69(8.9)	95(12.2)	113(14.6)	
	60-year-old and over	163(36.7)	58(13.1)	62(14.0)	22(5.0)	47(10.6)	92(20.7)	
Education N = 1651	< Primary school	168(51.9)	30(9.3)	39(12.0)	25(7.7)	28(8.6)	34(10.5)	< 0.001
	Primary school	222(43.4)	54(10.5)	61(11.9)	41(8.0)	60(11.7)	74(14.5)	
	Secondary school	162(37.5)	58(13.4)	70(16.2)	30(6.9)	51(11.8)	61(14.1)	
	High school	72(28.8)	33(13.2)	35(14.0)	21(8.4)	38(15.2)	51(20.4)	
	University/college	33(24.8)	18(13.5)	22(16.5)	11(8.3)	18(13.5)	31(23.3)	
Job status N = 1647	Employed full-time	383(42.1)	74(8.1)	138(15.2)	71(7.8)	126(13.9)	117(12.9)	< 0.001
	Employed part-time	130(50.6)	30(11.7)	33(12.8)	24(9.3)	20(7.8)	20(7.8)	
	Not employed	97(37.0)	46(17.6)	22(8.4)	23(8.8)	34(13.0)	40(15.3)	
	Retired/in school	43(19.6)	44(20.1)	34(15.5)	10(4.6)	14(6.4)	74(33.8)	
Province N = 1662	Thua Thien Hue	376(53.2)	31(4.4)	105(14.9)	46(6.5)	31(4.4)	118(16.7)	< 0.001
	Quang Tri	156(38.7)	28(6.9)	86(21.3)	18(4.5)	61(15.1)	54(13.4)	
	Khanh Hoa	126(22.8)	137(24.8)	37(6.7)	65(11.8)	104(18.8)	83(15.0)	
Residential area N = 1662	Urban	72(11.7)	154(24.9)	59(9.5)	69(11.2)	92(14.9)	172(27.8)	< 0.001
	Rural	586(56.1)	42(4.0)	169(16.2)	60(5.7)	104(10.0)	83(8.0)	
Self-rated Health N = 1660	Good	350(40.0)	108(12.4)	110(12.6)	62(7.1)	117(13.4)	127(14.5)	0.167
	Fair/poor	308(39.2)	88(11.2)	116(14.8)	67(8.5)	79(10.1)	128(16.3)	
Chronic health problems N = 1612	Yes	94(47.5)	13(6.6)	33(16.7)	16(8.1)	12(6.1)	30(15.2)	0.005
	No	541(38.3)	173(12.2)	191(13.5)	109(7.7)	184(13.0)	216(15.3)	
Time of affiliation N = 1628	Less than 6 months	61(30.5)	31(15.5)	30(15.0)	21(10.5)	27(13.5)	30(15.0)	< 0.001
	6 months to1 year	50(26.7)	39(20.9)	32(17.1)	13(7.0)	20(10.7)	33(17.6)	
	1–2 years	87(26.3)	41(12.4)	59(17.8)	38(11.5)	56(16.9)	50(15.1)	
	3–4 years	85(35.6)	31(13.0)	25(10.5)	22(9.2)	34(14.2)	42(17.6)	
	5 or more years	358(53.4)	49(7.3)	78(11.6)	32(4.8)	57(8.5)	97(14.5)	
Visited health the facility mainly because of a special medical problem? N = 1628	Yes	472(38.9)	143(11.8)	180(14.9)	102(8.4)	129(10.6)	186(15.3)	0.059
	No	170(40.9)	50(12.0)	46(11.1)	24(5.8)	61(14.7)	65(15.6)	
Government Health Insurance N = 1658	Yes	503(43.3)	169(14.5)	172(14.8)	44(3.8)	79(6.8)	195(16.8)	< 0.001
	No	152(30.8)	27(5.5)	56(11.4)	82(16.6)	117(23.7)	59(12.0)	
Affordable for health care last year N = 1509	Yes	109(39.6)	21(7.6)	47(17.1)	24(8.7)	38(13.8)	36(13.1)	0.052
	No	467(37.8)	159(12.9)	158(12.8)	95(7.7)	150(12.2)	205(16.6)	

*CHC* Commune health center, *PLC* Poly clinic, *DHC* District health center, *PVC* Private clinic, *PHM* Pharmacy store, *GVH* Government hospital

Table 1 also outlines the socio-demographic characteristics of the participants. Several characteristics of participants such as gender, age, educational level, job status, residential area, presence of chronic health problems and access to health insurance coverage have statistically significant associations with healthcare-seeking behaviours. Most notably, people with chronic health problems tend to choose CHCs more frequently as their usual source of care than those without (47.5%, *n* = 94 vs 38.3%, *n* = 541; *p* = 0.005). Similarly, those with health insurance were more likely to utilize CHCs than uninsured patients (43.3%, *n* = 503 vs 30.8%, *n* = 152; *p* < 0.001). Uninsured people, paying out-of-pocket for health care services, more frequently chose the private sector (private clinics or pharmacies) as their usual source of care. The three provinces had somewhat different utilization patterns: for instance, in Khanh Hoa Province, a higher percentage of people utilized the private sector than compared with the other provinces and use of polyclinics slightly outnumbered use of CHCs. Rural inhabitants in particular were more likely to utilize CHCs (56.1%, *n* = 586, *p* < 0.001), while urban dwellers preferred government hospitals, polyclinics and pharmacies (27.8%, *n* = 172 GVH; 24.9%, *n* = 154 PLC; 14.9%, *n* = 92 PHM) over CHCs (11.7%, *n* = 72) (p < 0.001).

### Quality of primary care in different types of health care facilities

**Fig. 3:**
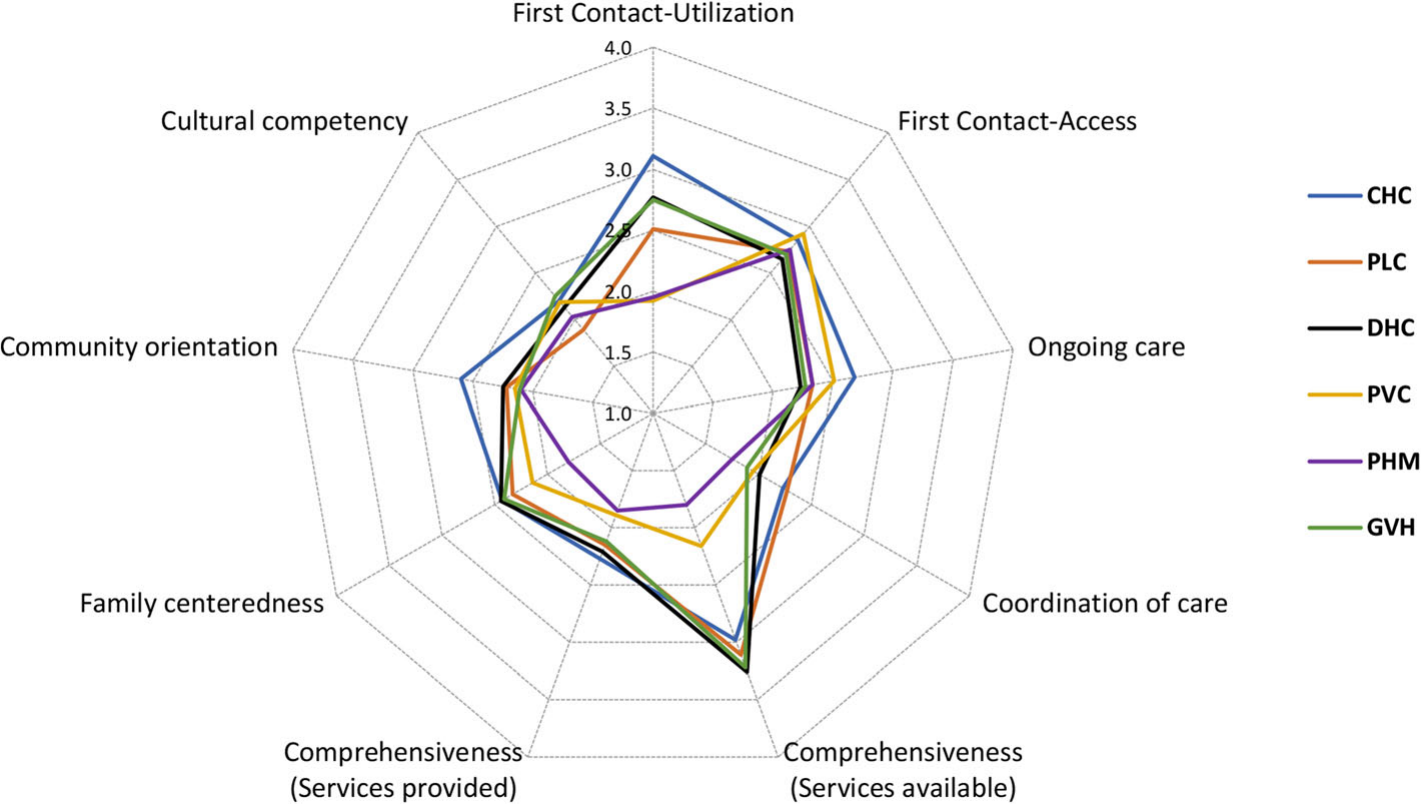
Quality of primary care in various domains by health care facilities

Table 2 (graphically presented in Fig. 3) shows the measures of each PCAT domain or subdomain by type of health care setting as well as total PCAT scores after adjusting for participants’ demographic characteristics. CHCs were associated with the highest quality in both total PCAT score (14.23, *p* < 0.001) and PCAT expanded score (21.07, *p* < 0.01) in comparison with other types of facilities. Regarding each attribute, CHCs were associated with the highest or second highest score for most attributes compared with other facilities, except for comprehensiveness of available services and cultural competency.

**Table 2:** Adjusted primary care attributes and total score reported by participants by type of health facility

Core and Derivative Domains	Subdomains		CHC	PLC	DHC	PVC	PHM	GVH	Significant differences
First contact	Utilization	Mean	2.92	2.34	2.64	1.95	1.93	2.56	CHC > all other*** PVC < all other *** but not significant with PHM PHM < all other *** except PVC PLC. DHC. GVH: no significant difference
		95 CI	(2.79–3.04)	(2.17–2.51)	(2.49–2.80)	(1.76–2.15)	(1.76–2.09)	(2.41–2.71)	
		n	607	175	217	116	191	234	
		Rank	1	4	2	5	6	3	
	Access	Mean	2.85	2.79	2.66	2.97	2.82	2.74	CHC > DHC*** DHC < CHC, PVC*** PVC > DHC***, GVH** No significant difference between PHM and others
		95 CI	(2.78–2.92)	(2.70–2.88)	(2.58–2.75)	(2.87–3.08)	(2.73–2.90)	(2.66–2.82)	
		n	609	177	215	115	192	236	
		Rank	2	4	6	1	3	5	
Ongoing care		Mean	2.56	2.32	2.15	2.50	2.31	2.22	CHC > all other***, but not significant with PVC PVC > DHC, GVH*** and PLC* DHC > PLC, PHM*
		95 CI	(2.49–2.63)	(2.23–2.41)	(2.06–2.23)	(2.39–2.60)	(2.23–2.40)	(2.14–2.30)	
		n	604	177	216	116	192	235	
		Rank	1	3	6	2	4	5	
Coordination of care		Mean	2.14	2.09	1.95	2.02	1.83	1.76	No significant difference between health care facilities
		95 CI	(1.91–2.37)	(1.76–2.42)	(1.68–2.22)	(1.68–2.37)	(1.52–2.14)	(1.49–2.03)	
		n	156	39	63	30	40	63	
		Rank	1	2	4	3	5	6	
Comprehensiveness	Available services	Mean	2.99	3.11	3.25	2.21	1.84	3.20	CHC < DHC, GVH*** and CHC > PVC, PHM*** DHC > CHC, PVC, PHM*** PVC > PHM, but<all others*** PHM < all others*** GVH > PHM, PVC, CHC***
		95 CI	(2.91–3.06)	(3.01–3.21)	(3.16–3.34)	(2.09–2.32)	(1.74–1.94)	(3.11–3.29)	
		n	598	167	215	111	180	228	
		Rank	4	3	1	5	6	2	
	Services provided	Mean	2.20	2.23	2.17	1.92	1.88	2.15	CHC > PVC*, PHM*** PVC < CHC*, DHC**, PLC* PHM < all others (CHC***. PLC***; DHC**GVH**) except PVC
		95 CI	(2.10–2.29)	(2.10–2.36)	(2.05–2.29)	(1.77–2.07)	(1.76–2.01)	(2.04–2.27)	
		n	598	175	212	112	190	230	
		Rank	2	1	4	5	6	3	
Family centeredness		Mean	2.45	2.34	2.45	2.21	1.89	2.42	PHM < all others (CHC, DHC, GVH***, PLC**), but not significant with PVC
		95 CI	(2.31–2.58)	(2.15–2.52)	(2.29–2.61)	(2.01–2.42)	(1.72–2.06)	(2.26–2.58)	
		n	599	174	215	117	186	229	
		Rank	1 (tie)	4	1 (tie)	5	6	3	
Community orientation		Mean	2.50	2.19	2.18	2.13	2.09	2.09	CHC > all others***, PLC**
		95 CI	(2.40–2.61)	(2.04–2.33)	(2.05–2.31)	(1.97–2.30)	(1.95–2.22)	(1.96–2.21)	
		n	602	169	215	109	179	226	
		Rank	1	2	3	4	5 (tie)	5 (tie)	
Cultural competency		Mean	2.12	1.87	2.14	2.17	2.00	2.20	PLC < GVH*
		95 CI	(1.98–2.26)	(1.68–2.06)	(1.97–2.31)	(1.96–2.39)	(1.82–2.19)	(2.04–2.37)	
		n	591	168	203	111	177	221	
		Rank	4	6	3	2	5	1	
Composite Scores	PCAT score	Mean	14.23	13.37	13.57	12.25	11.35	13.43	CHC > all others (PVC, PHM***; PLC, DHC, CHC**) PHM < PVC*, all others*** PVC < all other (CHC DHC, GVC***; PLC**) but except PHM
		95 CI	13.92–14.53)	(12.95–13.79)	(13.20–13.94)	(11.78–12.72)	(10.96–11.74)	(13.07–13.79)	
		n	604	174	216	113	190	233	
		Rank	1	3	2	5	6	4	
	PCAT expanded score	Mean	21.07	19.27	20.10	18.45	16.90	19.72	CHC > all others***, DHC* PHM < all others***, PVC* DHC < CHC* but >PVC** and PHM*** GVH < CHC*** but > PHM***
		95 CI	(20.57–21.57)	(18.59–19.94)	(19.49–20.71)	(17.68–19.22)	(16.26–17.54)	(19.13–20.31)	
		n	608	178	217	115	191	236	
		Rank	1	4	2	5	6	3	

*CHC* Commune health center, *PLC* Poly clinic, *DHC* District health center, *PVC* Private clinic, *PHM* Pharmacy store, *GVH* Government hospital

Scores were adjusted for gender, age, education level, job status, living area, chronic health problems, health insurance coverage and time affiliation with health facilities. Bonferroni post-hoc means test: significance indicated at *: *p* < 0.05; **: *p* < 0.01; ***: *p* < 0.001

In contrast, private clinics and pharmacies were generally rated most poorly on primary care domains, especially for first contact-utilization (1.95 PVC and 1.93 PHM), comprehensiveness of services available (2.21 PVC and 1.84 PHM), comprehensiveness of service provided (1.92 PVC and 1.88 PHM), family centeredness (2.21 PVC and 1.89 PHM) and in total PCAT score (12.25 PVC and 11.35 PHM) and total PCAT expanded score (18.45 PVC and 16.90 PHM). Private clinics achieved the highest score in first contact-access (2.97): significantly higher than district health centers (2.66, *p* < 0.001) and government hospitals (2.74, *p* = 0.003) but not significantly different from CHCs (2.85, *p* = 0.558).

With regards to the hospital setting, district health centers and government hospitals were evaluated as the second and the third highest in overall primary care quality, following CHCs in total PCAT scores (13.57 DHC and 13.43 GVH) and PCAT expanded scores (20.10 DHC and 19.72 GVH). Hospitals surpassed CHCs in consumers’ reports of the comprehensiveness of available services (3.25 DHC, 3.20 GVH, 2.99 CHC, p < 0.001), although they showed no significant difference in services provided (2.17 DHC, 2.15 GVH, 2.20 CHC, *p* = 1.000). Respondents who chose polyclinics as their usual source of care indicated the quality of these facilities to be better than private facilities but worse than other public facilities. Polyclinics performed quite well in comprehensiveness of services available (3.11) and first contact-access (2.79) but less so in other domains, especially in cultural competency (1.87).

## Discussion

Our research findings provide the first comprehensive and quantitative assessment of the quality of primary care at various types of health facilities in Vietnam. In this assessment, we found that CHCs play a central role in the effort to deliver high quality primary care to the population. CHCs not only had the highest utilization rate, but they also had the highest quality scores and overall highest primary care rankings in comparison with all other health facilities providing primary care services in Vietnam. Regarding specific domains, CHCs received the highest scores in first contact utilization, ongoing care, coordination, family centeredness and community orientation. CHCs were also scored highly by consumers in first contact access and comprehensiveness of services available compared with other health care facilities. A variety of factors may influence these scores, and CHCs may benefit by their design. For instance, access scores may in part reflect good geographic access resulting from the large number of CHCs distributed throughout the country. Specifically, the access to health facilities other than CHCs for rural residents may be more limited compared with urban residents, consistent with our finding that the utilization rate of CHCs by rural residents was higher than by urban residents. First contact utilization, continuity and comprehensiveness may be greater as CHCs are also the smallest health care units, closest to the community, and by mandate provide a wide variety of primary care services to care for people of all ages ranging from children to older people including pregnancy and maternal care. Moreover, because of their close relationship with and governmental responsibility for the care of local communities, CHCs incorporate some understanding of local context and culture, resulting in a strong community orientation.

Over the last two decades, Vietnam has invested more heavily in improving CHCs. Recently, the government has focused on improving both infrastructure and staff quality at the grassroots level, [30–33] and a previous study on public primary care centers in northern Vietnam demonstrated that CHCs have high capacity in delivering prevention and treatment services [15]. This investment in CHCs appears to be justified by our results, suggesting CHCs provide easily accessible, longitudinal and comprehensive care. Higher scores in these domains have been associated with better population-based health outcomes, suggesting government investment in CHCs is a rational and worthwhile strategy to improve overall health and well-being for all in Vietnam. This is consistent with previous research in China showing patient experiences with CHCs suggested equal or better primary care quality when compared with other health care providers (secondary and tertiary hospitals), supporting the appropriateness of the CHC delivery model in providing primary care to entire populations including the most vulnerable [6].

In contrast, the private sector, including both clinics and pharmacies, scored the lowest on overall quality of primary care provided in our study. The greatest deficits were seen in first contact utilization, coordination, comprehensiveness of services and family centeredness. Many of these domains may be impacted by a lack of integration between the public and private sectors. Private clinics, however, scored similarly to CHCs in first contact-access. This might be expected given first contact-access is a “customer service” attribute that may directly impact the profitability of private sector providers. Prior research has found that greater accessibility was one major reason that patients of private clinics in Hong Kong had better primary care experiences than those receiving care at general outpatient clinics [10]. Research in mainland China also demonstrated that primary care village clinics owned and managed by a private source scored higher in the PCAT domain of first contact-access when compared with those owned and managed by a hospital – however, they also received lower general scores for primary care quality [34]. On the other hand, a large review of 149 studies in 2003 found these studies increasingly report “no difference” in access between for-profit and non-profit providers in the U.S. Moreover, this review also pointed out that non-profit care was superior to for-profit on cost, quality and the amount of charity care provided in a majority of studies [35]. To promote improved quality in the private sector, building linkages to promote integration between private and public clinics could be useful to enhance the effectiveness of private health care facilities.

In Vietnam, medications - including antibiotics - are readily available at private pharmacies without a prescription. As a result, it is quite common that people will self-treat based on advice from prior provider encounters or family members or may just solicit advice from the pharmacist, and thus use a private pharmacy as their usual source of care rather than enduring long waits at more traditional primary care facilities. Despite this, patients’ experiences suggest pharmacies provide the lowest overall primary care quality as they lack a number of the essential elements and services associated with high quality primary care. Given this finding, the Vietnamese government may want to consider possible interventions to limit first-contact care-seeking behaviour by patients using pharmacies as the usual source of care without a doctor’s prescription.

In addition to their use of the private sector, many Vietnamese people bypass CHCs or other grassroots level facilities in preference of tertiary care hospitals with the expectation that such hospitals offer better quality due to more technological resources and a wider range of services. Medical literacy may also impact patient perception and choice of health facility if patients are uncertain about the severity of their condition or complexity of their care needs. Some research in other countries might support this expectation, such as in Malawi where work with the PCAT-Mw tool found that health centers scored lower than outpatient clinics in hospitals with regards to total primary care quality, first contact access and comprehensiveness of services available [36]. Our study in Vietnam, however, found that these perceptions are misguided as hospitals performed worse than CHCs in most attributes of primary care. While hospitals rated better in comprehensiveness of available services than CHCs, they scored more poorly in all other domains including the comprehensiveness of services provided. Our finding is consistent with other existing data from China and U.S. suggesting that hospitals and subspecialists are more likely to provide lower quality primary care than trained frontline providers [7, 11].

This study has several limitations. First, the head of household and another adult member were interviewed without a random sampling method within the household, leading to the possibility of some unintentional bias in the collection of responses. The failure to record precise non-response rates also introduces some lack of clarity about the degree of potential bias in our findings. Secondly, the number of participants from certain health facilities such as polyclinics, private clinics and pharmacies were quite small in comparison with the number attending CHCs, and therefore may not allow for the most accurate assessment of their consumers. Our study also is not designed to determine the specific service delivery aspects and activities within each type of facility that may lead to these findings, such as the inclusion of trained family physicians or the presence of specific equipment or medications. Because our sample was limited only to consumers of those communes with a physician working in the local CHC, we also cannot determine if the quality of primary care would be the same in those CHCs staffed without a physician.

## Conclusions

Despite these limitations, this study provides useful insights for policy-makers in low and middle-income countries as they seek to determine where to best incentivize and direct patient utilization of primary care services. The high quality of primary care services offered in CHCs compared with other facilities gives Vietnam ample reason to promote greater use of them. The typical pattern of self-pay patients bypassing CHCs in search of better quality care at hospitals appears to be misguided in Vietnam, and the government may want to consider more substantial efforts to alert the public to these misperceptions. Given the higher quality of primary care services offered at CHCs coupled with the increased availability and utilization by those with non-communicable diseases, low income and in hard-to-reach rural areas, CHCs also seem likely to have the most substantial effect on reducing those health inequities that can be improved by primary care. More study is needed, however, populations may benefit most by building and strengthening grassroots networks of community-based health centers as the most effective solution for overcrowding at upper level hospitals while simultaneously providing better overall health outcomes.

## Additional file

Additional file 1: Population denstity of study area. (PDF 5133 kb)

## Abbreviations

CHC: Commune health center
CHCs: Commune health centers
DHC: District health center
GVH: Government hospitals
PCAT: Primary Care Assessment Tool
PCAT-AE: Primary Care Assessment Tool adult consumer expanded version
PHM: Pharmacy store
PLC: Polyclinic
PVC: Private clinic
VN PCAT-AE: Vietnamese Primary Care Assessment Tool adult consumer expanded version
