# Supplementary material for: Patient experiences of primary care quality amongst different types of health care facilities in central Vietnam
Source: BMC Health Serv Res. 2019 May 2;19:275. doi: 10.1186/s12913-019-4089-y (PMC6498623; doi:10.1186/s12913-019-4089-y)
Supplement: Supplementary file 1 — Population denstity of study area. (PDF 5133 kb) [file 12913_2019_4089_MOESM1_ESM.pdf]

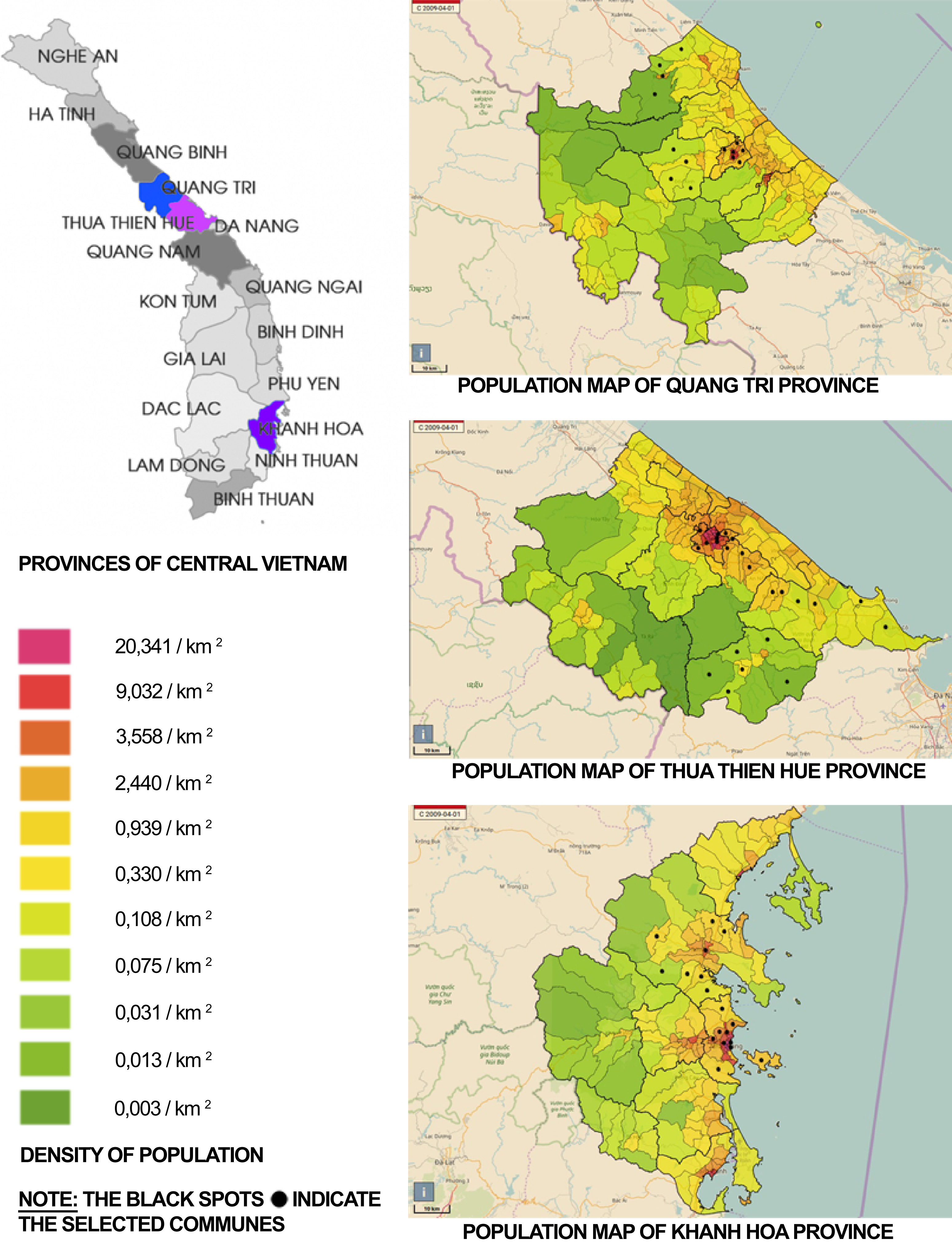

Figure. Population density of study area.

Data was extracted from: https://www.citypopulation.de/
